# Pharmacokinetics and Safety of Single-Dose Sotrovimab in High-Risk Children and Adolescents With Mild-to-Moderate COVID-19

**DOI:** 10.1093/jpids/piaf027

**Published:** 2025-03-27

**Authors:** Jennifer Moore, Daren Austin, Alicia Aylott, Jerzy Daniluk, Leah A Gaffney, Marjan Hezareh, Ahmed Nader, Nadia Noormohamed, Charlene Parado, Amanda Peppercorn, Yessica Sachdeva, Scott Segal, Klaudia Steplewski, Phillip J Yates, Jill Walker, Andrew Skingsley

**Affiliations:** GSK, London, United Kingdom; GSK, London, United Kingdom; GSK, Stevenage, United Kingdom; GSK, Warsaw, Poland; Vir Biotechnology, Inc., San Francisco, CA, United States; GSK, London, United Kingdom; GSK, Collegeville, PA, United States; GSK, Collegeville, PA, United States; GSK, Mississauga, Ontario, Canada; GSK, Cambridge, MA, United States; Arizona Clinical Trials, Chandler, AZ, United States; GSK, Collegeville, PA, United States; GSK, Collegeville, PA, United States; GSK, Stevenage, United Kingdom; GSK, San Francisco, CA, United States; GSK, London, United Kingdom

**Keywords:** COVID-19, pediatrics, pharmacokinetics, safety, sotrovimab

## Abstract

Sotrovimab was well tolerated by children/adolescents (6 to <18 years) with mild-to-moderate COVID-19 at high risk of progression to severe disease. Pharmacokinetic parameters in adolescents (12 to <18 years) were generally similar to those reported in adult studies of sotrovimab.

Previous studies have detailed the efficacy/safety and pharmacokinetics (PK) of sotrovimab in adult patients with mild-to-moderate COVID-19.^1-3^ The Phase 2b COMET-PACE study assessed the PK, pharmacodynamics (PD), and tolerability of sotrovimab in children and adolescents (aged <18 years) with COVID-19 (full eligibility criteria in Supplementary Table 1), intended as part of a PK-bridging approach. The study design included 2 cohorts (*N* = 36 planned for each), with Cohort A receiving intravenous (IV) sotrovimab and Cohort B receiving intramuscular (IM) sotrovimab (Supplementary Figure 1); however, enrollment was paused in March 2022 due to decreased in vitro neutralization of sotrovimab against circulating SARS-CoV-2 variants during this period (BA.2).^4^ Consequently, no participants received IM dosing (Part B). The study was terminated in June 2023 for programmatic reasons.

Primary objectives were to evaluate the PK, safety, and tolerability of sotrovimab through day 29 and week 36. Secondary objectives were COVID-19 disease progression, change in SARS-CoV-2 viral load over time, change in laboratory parameters, and occurrence of disease-related events and multisystem inflammatory syndrome (MIS-C) through day 29. No formal statistical significance tests were performed.

Eight (out of a planned 36) participants received treatment with sotrovimab IV (Supplementary Figure 2). Two participants (both 6 to <12 years; <40 kg) received sotrovimab 250 mg IV and the remaining 6 (*n* = 5 aged 12 to <18 years, *n* = 1 aged 6 to <12 years; all >40 kg) received sotrovimab 500 mg IV. Median duration of post-dose follow-up was 253 days. Median age of participants was 13.5 years; none were aged <6 years and all had ≥1 predefined risk factor for COVID-19 progression (Supplementary Table 2).

Sotrovimab serum-concentration results are shown in Figure 1 and Supplementary Table 3. Comparison of PK parameters with the final sotrovimab model (see Supplementary Material) revealed comparable exposures between adolescents (12 to <18 years) and adults, and higher exposures in the 6 to <12 years age group as compared to adults (geometric mean AUC_0–D29_ 1.7-fold and *C*_max_ 1.4-fold higher; Table 1). PK results in the 6 to <12 years age group should be interpreted with caution, however, given the small number of assessable participants (*n* = 1) for some parameters (Table 1; Supplementary Table 4).

**Table 1.** Summary of Exposure and PK Parameters by Age Group (PK Population)

Parameter	Statistics	6 to <12 years	12 to <18 years	Total	Sotrovimab 500 mg IV adults
	n	3	5	8	1188
AUC_0–inf_, μg*day/mL	Mean (SD)	6040.1 (477.7)	5091.0 (1241.1)	5421.1 (1130.3)	5253.6 (1643.2)
	Geometric mean (geometric %CV)	6023.0 (7.91)	4928.8 (24.4)	5284.8 (20.9)	4961.7 (37.4)
	Median (5^th^, 95^th^ percentile)	5742.0 (5716, 6726)	5379.1 (3182, 6444)	5716.9 (3182, 6726)	5204 (2568, 8116)
AUC_0–D29_, μg*day/mL	Mean (SD)	2711 (571.5)	1956.8 (363.2)	2292.2 (588.7)	1637.1 (444.9)
	Geometric mean (geometric %CV)	2664.0 (21.1)	1924.3 (18.6)	2223.6 (25.7)	1563.6
	Median (5th, 95th percentile)	2820.8 (2088, 3182)	2089.6 (1466, 2181)	2105.6 (1597, 3182)	1622
*C*_max_ (μg/mL)	Mean (SD)	248.8 (69.5)	198.6 (42.9)	216.1 (58.1)	188.7 (82.3)
	Geometric mean (geometric %CV)	239.8 (27.9)	193.3 (21.6)	208.4 (26.9)	170.1
	Median (5th, 95th percentile)	239.3 (170, 339)	209.4 (122, 249)	213.4 (122, 339)	188.5
*C*_D29_ (μg/mL)	Mean (SD)	68.0 (10.4)	48.0 (9.9)	56.9 (14.2)	42.0 (12.9)
	Geometric mean (geometric %CV)	67.4 (15.3)	47.0 (20.7)	55.1 (24.9)	–
	Median (5th, 95th percentile)	69.9 (57, 77)	51.8 (34, 54)	54.9 (38, 77)	41.48
CL (L/day)	Mean (SD)	0.05 (0.01)	0.10 (0.03)	0.09 (0.04)	0.10 (0.05)
	Geometric mean (geometric %CV)	0.05 (27.6)	0.10 (28.5)	0.08 (41.0)	0.10 (35.0)
	Median (5th, 95th percentile)	0.04 (0.04, 0.07)	0.09 (0.07, 0.15)	0.08 (0.04, 0.16)	0.09 (0.06, 0.17)
Steady-state volume of distribution (L)	Mean (SD)	2.8 (0.48)	6.22 (1.5)	5.02 (2.1)	8.40 (6.1)
	Geometric mean (geometric %CV)	2.7 (17.4)	6.07 (23.8)	4.59 (41.1)	7.88 (30.7)
	Median (5th, 95th percentile)	2.87 (2.2, 3.3)	5.78 (4.6, 8.9)	5.7 (2.2, 9.0)	7.7 (5.3, 12.5)
*t*_1/2_ (days)	Mean (SD)	37.9 (6.1)	44.8 (9.9)	42.4 (9.3)	61.4 (9.8)
	Geometric mean (geometric %CV)	37.5 (16.1)	43.6 (22.0)	41.4 (21.9)	60.7 (15.7)
	Median (5th, 95th percentile)	35.7 (32, 46)	44.2 (27, 57)	43.3 (29, 57)	61.2 (48, 75)

Abbreviations: %CV, coefficient of variation expressed as a percent; AUC_0–inf_, area under the curve extrapolated to infinity; AUC_0–D29_, area under the curve from time 0 to day 29; C_D29_, serum concentration at day 29; CL, clearance; *C*_max_, peak serum concentration; IV, intravenous; PK, pharmacokinetics; SD, standard deviation; *t*_1/2_, apparent terminal phase half-life.

Due to the limited number of pediatric participants, a previously developed population PK model for sotrovimab was utilized to generate individual post-hoc parameters for each participant.

**Figure 1.**
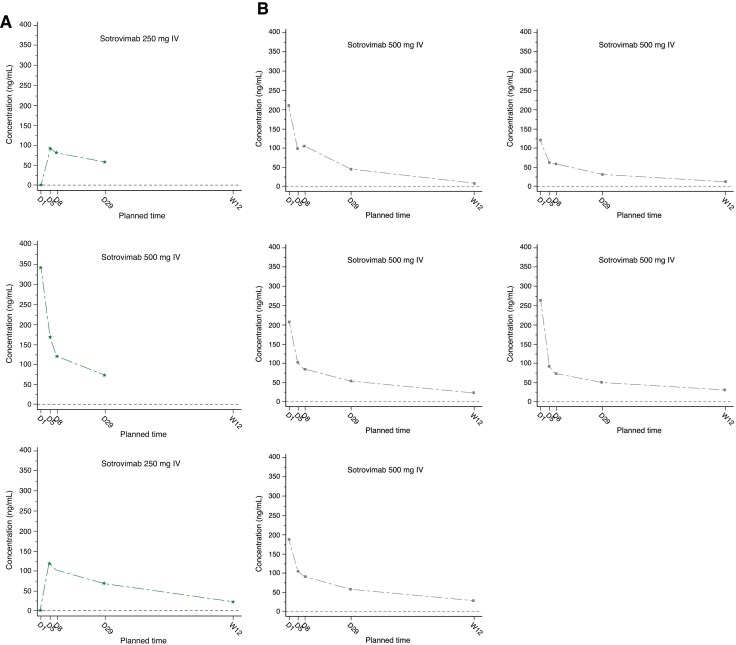
Individual sotrovimab serum concentration–time plots (µg/mL) through week 12 (PK principal stratum population). (A) 6 to <12 years (*n* = 3^a^); (B) 12 to <18 years (*n* = 5). D, day; IV, intravenous; PK, pharmacokinetics; W, week. Lower limit of quantification = 0.1 µg/mL. ^a^Two participants in the 6 to <12 years age group (both receiving sotrovimab 250 mg IV) had day 1 end-of-infusion values as nonqualified and were imputed with a zero value.

Six post-dose adverse events (AEs) in 2 participants were reported through day 29, and 9 in 4 participants were reported up to week 36; none were considered to be treatment-related, and all were of Grade 1 or 2 severity (Supplementary Table 5). There were no treatment-related changes in laboratory values, vital signs, or electrocardiograms. There were no reports of serious AEs or AEs leading to interrupted/incomplete infusion, MIS-C, infusion-related reactions occurring within 24 hours post-dose, hypersensitivity reactions at any time post-dose, events suggestive of antibody-dependent enhancement, or immunogenicity-related AEs. Through day 29, no participants reported progression of COVID-19 or development of severe and/or critical respiratory COVID-19 requiring supplemental oxygen. There were no deaths reported through week 36. One participant had treatment-emergent antidrug antibodies (not neutralizing).

Viral-load data showed a decrease from baseline over time in mean values at all time points through day 29 (Supplementary Table 6; Supplementary Figure 3). Two (25%) participants (both in the 6 to <12 years age group) met the criteria for viral rebound; both were infected with Omicron BA.1.1, and no treatment-emergent substitutions in the sotrovimab epitope were observed. All participants had an undetectable viral-load result on day 29.

Some limitations should be acknowledged. There was no control arm, and early termination of enrollment resulted in a small sample size (enrolled from only 2 centers), both of which limit the interpretation of the findings. Only 1 participant in the 6 to <12 years age group had a quantifiable sample for analysis of some PK parameters. In addition, all eight participants enrolled were infected with Omicron BA.1 or BA1.1 subvariants. Of the small number of enrolled participants, none were aged <6 years.

In conclusion, a single weight-based IV infusion of sotrovimab (250 or 500 mg) given to children/adolescents (6 to <18 years) with mild-to-moderate COVID-19 at high risk of progression to severe disease was well tolerated. Among the 8 participants enrolled, no new safety concerns related to sotrovimab were identified and COVID-19-related clinical outcomes were seemingly favorable. PK parameters in the adolescent population (12 to <18 years) were generally similar to those reported previously in adult studies of sotrovimab, indicating that the current weight-based dosing appears appropriate for adolescents. The exposure equivalence and safety of sotrovimab have not been established in children aged <12 years or weighing <40 kg.

## Supplementary Material

piaf027_suppl_Supplementary_Materials

## Data Availability

Please contact the corresponding author for requests for access to anonymized subject-level data.

## Medical Writing, Editorial, and Other Assistance

Editorial support (in the form of writing assistance, including preparation of the draft manuscript under the direction and guidance of the authors, collating and incorporating authors’ comments for each draft, assembling tables, grammatical editing, and referencing) was provided by Tony Reardon of Luna, OPEN Health Communications, in accordance with Good Publication Practice (GPP) guidelines (www.ismpp.org/gpp-2022). The support was funded by GSK.

## Notes

ClinicalTrials registration: NCT05124210
