# Supplementary material for: Pharmacokinetics and Safety of Single-Dose Sotrovimab in High-Risk Children and Adolescents With Mild-to-Moderate COVID-19
Source: J Pediatric Infect Dis Soc. 2025 Mar 27;14(5):piaf027. doi: 10.1093/jpids/piaf027 (PMC12084821; doi:10.1093/jpids/piaf027)
Supplement: piaf027_suppl_Supplementary_Materials [file piaf027_suppl_supplementary_materials.docx]

# Supplementary Appendix

**Statistical analyses**

Due to the small number of participants who completed study assessments (N = 8), the final sotrovimab population PK model was used to generate individual post-hoc exposures and derived PK parameters for each participant. Serum concentration data were added to the final population PK dataset that included 1891 adult and adolescent participants (n = 30 aged ≤18 years) with COVID-19 enrolled in previous sotrovimab clinical studies (COMET-ICE [NCT045450060], COMET-PEAK [NCT04779879], BLAZE-4 [NCT04634409], and COMET-TAIL [NCT04913675]), and 38 healthy individuals enrolled in GSK Pharma Study 217653. Post-hoc derived exposure and PK parameters using non-compartmental analyses (NCA) were also calculated and compared against PK parameters from the adult population.

The virology analysis set comprised all participants exposed to sotrovimab who had a quantifiable SARS-CoV-2 viral-load measurement at baseline. The PK Principal Stratum population included all participants in the PK analysis set (i.e., all participants exposed to study intervention who had at least one non-missing PK assessment) who would be able to complete the IV dose. The safety analysis comprised all participants exposed to the study intervention. Missing data were not imputed.

### **Supplementary Table 1. Inclusion and Exclusion Criteria**

| Inclusion criteria | Exclusion criteria |
| --- | --- |
| - Male and female participants of 32 weeks estimated gestational age, day of life 0 to <18 years of age inclusive, at either the time of participant’s signed assent (if age-appropriate) or parent(s)/legally authorized representative signing the informed consent - Have mild-to-moderate COVID-19, defined by:   - A positive SARS-CoV-2 test result by any validated qRT-PCR or other NAAT, AND   - SpO_2_ ≥94% on room air,^a^ AND   - Have one or more of the following symptoms: fever, chills, cough, sore throat, malaise, headache, joint or muscle pain, change in smell or taste, vomiting, diarrhea, shortness of breath, poor appetite or poor feeding, nasal congestion/runny nose, lethargy, AND   - ≤7 days from onset of symptoms to dosing day (Day 1) - Participants at risk of disease progression with ≥1 of the following criteria:   - Age <1 year   - Diabetes mellitus   - Genetic or metabolic diseases   - Obesity (BMI [kg/m^2^] ≥95th percentile for age and sex based on local/CDC/WHO growth charts for children ≥2 years of age)   - Cardiovascular disease   - Sickle cell disease   - Pulmonary disease   - Neurologic disease   - Immunosuppressed^b^   - Baseline medical complexity^c^ - Body weight within the following range:   - Preterm infants and term newborn infants: ≥2 kg   - Children 2 years to <18 years: BMI ≥5^th^ percentile for age based on local/CDC/WHO growth charts | - Pregnant or breastfeeding - Currently hospitalized, or judged by the investigator as likely to require hospitalization in the next 24 hours, due to severe or critical COVID-19^d^ - Respiratory rate:   - 0–6 months: >60 breaths/minute (min)   - 6 months to 5 years: >30 breaths/min   - 6 years to <18 years: >20 breaths/min - Shortness of breath at rest or respiratory distress - Shock (septic, neurogenic, anaphylactic, cardiogenic, or hypovolemic shock; systemic inflammatory response syndrome) - Multiorgan dysfunction - Participants who, in the judgement of the investigator, were likely to die in the next 7 days - Multisystem inflammatory syndrome - Post-conceptional age <32 completed weeks at time of Screening - History of sudden infant death or unexplained death in a sibling - For Cohort B only: any condition that would prohibit receipt of IM injections in the investigator’s opinion (e.g., coagulation disorder, bleeding diathesis, or thrombocytopenia [platelet count <50 000/mm^3^]) - Prior, current, or planned future use of any of the following treatments during the study period: COVID-19 convalescent plasma, mAbs against SARS-CoV-2 (e.g., casirivimab/imdevimab), intravenous immunoglobulin for any indication, or dexamethasone specifically for treatment of COVID-19 - Current use of COVID-19 treatment (authorized, approved, or investigational) - The following exclusions related to use of an authorized or approved vaccine for SARS-CoV-2:   - Receipt of any authorized or approved vaccine for SARS-CoV-2 within 48 hours prior to dosing   - Planned use of any authorized or approved vaccine for SARS-CoV-2 within 90 days of study-drug administration   - Receipt of any non-SARS-CoV-2 vaccines within 14 days (for non-live vaccines) or 28 days (for live vaccine) of Screening - Participated, or currently participating, in a clinical research study evaluating COVID-19 convalescent plasma, mAbs against SARS-CoV-2 (e.g., casirivimab/imdevimab), or intravenous immunoglobulin within 3 months or within 5 half-lives of the investigational product (whichever is longer) prior to the Screening visit - Participated, or currently participating, or planned to participate during the study period in a clinical research study evaluation of any authorized, approved, or investigational vaccine for SARS-CoV-2 - Currently enrolled in another clinical study - Infants <24 weeks of age: maternal receipt of intravenous immunoglobulin, SARS-CoV-2-directed convalescent plasma. or SARS-CoV-2-directed mAb(s) within 3 months prior to birth or within 5 half-lives of the investigational product (whichever is longer) - Participants who, in the judgment of the investigator, were unlikely or unable to comply with the requirements of the protocol through the end of the study - Known hypersensitivity to any constituent present in the investigational product |

Abbreviations: BiPAP, bilevel positive airway pressure; BMI, body mass index; CDC, Centers for Disease Control and Prevention; COVID-19, coronavirus 2019; CPAP, continuous positive airway pressure; mAb, monoclonal antibody; NAAT, nucleic acid amplification test; qRT-PCR, quantitative reverse transcriptase polymerase chain reaction; SARS-CoV-2, severe acute respiratory syndrome coronavirus-2; SpO_2_, oxygen saturation; WHO, World Health Organization.

^a^Some participants may have been on baseline oxygen supplementation or respiratory support (CPAP, BiPAP, or ventilator-dependence) prior to contracting COVID-19. Others, such as participants with some unrepaired congenital heart disease, may have had baseline SpO_2_ that was <94% on room air prior to contracting COVID-19. If a participant could maintain his/her baseline SpO_2_ while on baseline oxygen supplementation (including room air) and baseline respiratory support (modality, pressures, and frequency of use), they could be included in the study.

^b^Use of systemic corticosteroids was included, defined by dose ≥0.5 mg/kg/day or ≥20 mg/day prednisone equivalents (whichever dose was the lower of the two) taken for ≥2 weeks.

^c^Gastrostomy- or jejunostomy-dependence, parenteral nutrition dependence, tracheostomy-dependence, baseline oxygen requirement, use of CPAP/BiPAP/ventilator support.

^d^If the infant had been hospitalized due to other reasons (e.g., prematurity) or was hospitalized for reason of administering the study intervention, they could be included in the study.

**Supplementary Table 2. Baseline Demographics and Disease Characteristics (Safety Population)**

|  | Sotrovimab IV  (6 to <12 years)  (n = 3) | Sotrovimab IV  (12 to <18 years)  (n = 5) | Sotrovimab IV  Total  (N = 8) |
| --- | --- | --- | --- |
| Sex, n  Female  Male | 1  2 | 4  1 | 5  3 |
| Age, years^a^  Mean (SD)  Median (min, max) | 9.7 (0.6)  10.0 (9, 10) | 14.2 (1.1)  14.0 (13, 16) | 12.5 (2.5)  13.5 (9, 16) |
| Ethnicity, n  Hispanic or Latino  Not Hispanic or Latino | 1  2 | 1  4 | 2  6 |
| Race, n  Black/African American  White | 0  3 | 1  4 | 1  7 |
| BMI, kg/m^2^  Mean (SD)  Median (min, max) | 18.9 (3.7)  18.4 (15.5, 22.8) | 31.1 (9.8)  34.7 (20.2, 41.9) | 26.5 (10.0)  21.9 (15.5, 41.9) |
| Risk factors for COVID-19 progression,^b^ n  Any condition  Obesity  Diabetes mellitus  Cardiovascular disease  Pulmonary disease  Immunosuppressed  Neurologic disease | 3  1  1  1  1  1  1 | 5  3  2  1  1  0  0 | 8  4  3  2  2  1  1 |
| Symptoms present, n  Any symptom  Cough | 3  3 | 5  4 | 8  7 |
| Congestion or runny nose | 3 | 2 | 5 |
| Headache | 1 | 4 | 5 |
| Muscle aches (myalgia)^c^ | 1 | 4 | 5 |
| Sore throat^c^ | 2 | 3 | 5 |
| Chills | 1 | 3 | 4 |
| Fatigue | 1 | 3 | 4 |
| Fever | 2 | 2 | 4 |
| Malaise | 0 | 2 | 2 |
| Nausea^c^ | 2 | 0 | 2 |
| Vomiting | 2 | 0 | 2 |
| Abdominal pain^c^ | 1 | 0 | 1 |
| Chest pain^c^ | 1 | 0 | 1 |
| Loss of smell^c^ | 1 | 0 | 1 |
| Shortness of breath | 0 | 1 | 1 |
| Skin rash | 1 | 0 | 1 |
| Symptom duration, n (%)  ≤3 days  4–5 days  6–7 days | 2  1  0 | 3  1  1 | 5  2  1 |
| Baseline SARS-CoV-2 viral load (log_10_ copies/mL) in nasal mid-turbinate sample^d,e^  n  Mean (SD)  Median (min, max)  ≥2.08 to <4, n  ≥4 to <5, n  ≥5 to <6, n  ≥6 to <7, n | 2  5.085 (0.2475)  5.085 (4.91, 5.26)  0  1  1  0 | 5  4.712 (1.0668)  4.180 (3.75, 6.34)  1  2  1  1 | 7  4.819 (0.8956)  4.910 (3.75, 6.34)  1  3  2  1 |

Abbreviations: BMI, body mass index; COVID-19, coronavirus disease 2019; IV, intravenous; max, maximum; min, minimum; SARS-CoV-2, severe acute respiratory syndrome coronavirus-2; SD, standard deviation.

Participants may occur more than once in the list of risk factors and the list of symptoms present. Relevant medical history within the last 3 years included baseline COVID-19 symptoms, comorbidities, and tobacco use.

^a^Age calculation uses the last day of the month and calculates age relative to screening date.

^b^Medical conditions present as risk factors at screening.

^c^Symptoms only collected for age 5 years and up.

^d^Negative and <2.08 log_10_ copies/mL viral-load samples were imputed as: 0.5x120 copies/mL=60 copies/mL=1.78 log_10_ copies/mL. One participant was missing a quantifiable sample at baseline. Percentages are based on the number of non-missing results.

^e^Three participants (n = 1 aged 6 to <12 years; n = 2 aged 12 to <18 years) received COVID-19 vaccination (Pfizer, two doses).

### **Supplementary Table 3.** **Sotrovimab Serum Concentration-time Data (µg/mL) Through Week 12 (PK Principal Stratum Population)**

**A) By age group**

| Planned time | 6 to <12 years (n = 3^a^)  Sotrovimab 250 mg IV (n = 2) and 500 mg IV (n = 1) | | | | 12 to <18 years (n = 5)  Sotrovimab 500 mg IV | | | |
| --- | --- | --- | --- | --- | --- | --- | --- | --- |
|  | n | Mean (SD) | 95% CI | Median (range) | n | Mean (SD) | 95% CI | Median (range) |
| Day 1 | 3^a^ | 114.32 (–) | – | 0.00 (0, 342.96) | 5 | 197.72 (51.77) | (133.44-261.99) | 207.63 (120.62, 263.86) |
| Day 5 | 3 | 125.26 (38.62) | (29.32-221.20) | 118.38 (90.54, 166.86) | 5 | 90.91 (16.94) | (69.88-111.94) | 98.49 (61.78, 102.57) |
| Day 8 | 3 | 99.02 (18.88) | (52.11-145.93) | 99.58 (79.87, 117.63) | 5 | 81.41 (17.61) | (59.54-103.28) | 82.65 (58.12, 105.10) |
| Day 29 | 3 | 66.49 (7.34) | (48.26-84.72) | 67.91 (58.54, 73.01) | 5 | 47.00 (9.62) | (35.05-58.95) | 50.50 (31.16, 55.71) |
| Week 12 | 1 | 21.92 (–) | – | 21.92 (21.92, 21.92) | 5 | 19.70 (8.42) | (9.25-30.16) | 21.58 (9.26, 28.68) |

**B) Overall (n = 8)**

| Planned time | n | Mean (SD) | 95% CI | Median (range) | %CV |
| --- | --- | --- | --- | --- | --- |
| Day 1 | 8^b^ | 166.44 (120.82) | (65.44-267.45) | 196.79 (0.00, 342.96) | 72.59 |
| Day 5 | 8 | 103.79 (30.10) | (78.62-128.95) | 99.82 (61.78, 166.86) | 29.00 |
| Day 8 | 8 | 88.02 (19.03) | (72.10-103.93) | 85.72 (58.12, 117.63) | 21.62 |
| Day 29 | 8 | 54.31 (13.04) | (43.41-65.21) | 54.03 (31.16, 73.01) | 24.01 |
| Week 12 | 6 | 20.07 (7.58) | (12.11-28.03) | 21.75 (9.26, 28.68) | 37.78 |

Abbreviations: %CV, coefficient of variation expressed as a percent; CI, confidence interval; IV, intravenous; PK, pharmacokinetics; SD, standard deviation.

^a^Two participants in the 6 to <12 years age group (both receiving sotrovimab 250 mg IV) had Day 1 end-of-infusion values below Lower Limit of Quantification (<0.1 μg/mL) and concentration value imputed to zero at Day 1 timepoint. If these two values are excluded, the Mean and Median values would be 342.96 and 342.96 μg/mL, respectively.

^b^Two participants in the 6 to <12 years age group (both receiving sotrovimab 250 mg IV) had Day 1 end-of-infusion values below Lower Limit of Quantification (<0.1 μg/mL) and concentration value imputed to zero. If these two values were excluded, the post-hoc results for Day 1 would be Mean (SD) 221.92 (75.231), 95% CI 142.97-300.87, Median 209.07 (120.62-342.96) and %CV 33.9 μg/mL.

**Supplementary Table 4. Summary of Post-hoc Derived Sotrovimab Serum PK Parameters Through Week 12 (PK Principal Stratum Population): Non-compartmental Analysis**

| Parameter | Statistics | 6 to <12 years  (n = 3) |  | 12 to <18 years  (n = 5) | Total  (n = 8) | Sotrovimab  500 mg IV adult (PK population) |
| --- | --- | --- | --- | --- | --- | --- |
| AUC_0–inf_, μg*day/mL | n | 1 |  | 5 | 6 | 1188 |
|  | Mean (SD) | 5865.3 (–) |  | 5349.6 (1421.0) | 5435.5 (1288.3) | 5253.6 (1643.2) |
|  | Median (min, max) | 5865.3 (5865.3, 5865.3) |  | 5635.5 (3230.3, 6782.6) | 5750.4 (3230.3, 6782.6) | 5204 (2568, 8116)^a^ |
| AUC_0–D29_, μg*day/mL | n | 1 |  | 5 | 6 | 1188 |
|  | Mean (SD) | 3190.4 (–) |  | 2041.2 (366.8) | 2232.7 (572.5) | 1637.1 (444.9) |
|  | Median (min, max) | 3190.4 (3190.4, 3190.4) |  | 2166.2 (1409.6, 2318.6) | 2211.6 (1409.6, 3190.4) | 1622 (925, 2376)^a^ |
| C_max_ (μg/mL) | n | 1 |  | 5 | 6 | 1188 |
|  | Mean (SD) | 343.0 (–) |  | 197.7 (51.8) | 221.9 (75.2) | 188.7 (82.3) |
|  | Median (min, max) | 343.0 (343.0, 343.0) |  | 207.6 (120.6, 263.9) | 209.1 (120.6, 343.0) | 188.5 (78.4, 309.8)^a^ |
| C_D29_ (μg/mL) | N | 3 |  | 5 | 8 | 1188 |
|  | Mean (SD) | 66.5 (7.3) |  | 47.0 (9.6) | 54.3 (13.0) | 42.0 (12.9) |
|  | Median (min, max) | 67.9 (58.5, 73.0) |  | 50.5 (31.2, 55.7) | 54.0 (31.2, 73.0) | 41.5 (21.3, 64.2)^a^ |
| CL (mL/day) | N | 1 |  | 5 | 6 | 1927 |
|  | Mean (SD) | 42.6 (–) |  | 100.3 (32.9) | 90.7 (37.7) | 101 (50) |
|  | Median (min, max) | 42.6 (42.6, 42.6) |  | 88.7 (73.7, 154.8) | 83.6 (42.6, 154.8) | 91 (60, 170)^a^ |
| Steady-state volume of distribution (L) | n | 1 |  | 5 | 6 | 1927 |
|  | Mean (SD) | 1.3 (–) |  | 5.0 (1.3) | 4.4 (1.9) | 8.4 (6.1) |
|  | Median (min, max) | 1.3 (1.3, 1.3) |  | 4.7 (3.3, 6.7) | 4.7 (1.3, 6.7) | 7.7 (5.3, 12.5)^a^ |
| t_1/2_ (days) | n | 3 |  | 5 | 8 | 1927 |
|  | Mean (SD) | 32.9 (9.1) |  | 40.8 (11.9) | 37.8 (11.0) | 61.4 (9.8) |
|  | Median (min, max) | 34.6 (23.0, 44.1) |  | 40.5 (27.1, 58.1) | 37.6 (23.0, 58.1) | 61.2 (47.8, 75.1)^a^ |

Abbreviations: AUC_0–D29_, area under the curve from time 0 to Day 29; AUC_0–inf_, area under the curve extrapolated to infinity; C_D29_, serum concentration at Day 29; CL, clearance; C_max_, peak serum concentration; IV, intravenous; PK, pharmacokinetics; SD, standard deviation; t_½_, apparent terminal phase half-life.

Two participants aged 6 to <12 years were excluded from these summaries due to missing end-of-infusion sample. The remaining participant received 500 mg.

^a^Adult data show median (5^th^, 95^th^ percentile).

## Supplementary Table 5. Summary of Post-dose^a^ AEs at Week 36 (Safety Population)

| Age group | AE | Time since dose | Maximum grade^b^ | Outcome | Medical conditions of relevance to the AE |
| --- | --- | --- | --- | --- | --- |
| 6 to <12 years^c^ | Blood creatinine increase | 8 days | Grade 2 | Recovered/resolved  (duration = 7 days) | *Current:*  immunocompromised state,  graft-versus-host  disease, cytopenias    *Past:* hematological malignancy |
|  | Non-COVID-19 viral complication associated with immunosuppression | 9 days | Grade 2 | Not recovered/not resolved |  |
|  | Alanine aminotransferase increase | 29 days | Grade 2 | Not recovered/not resolved |  |
|  | Aspartate aminotransferase  increase | 29 days | Grade 2 | Not recovered/not resolved |  |
|  | Worsening of graft-versus-host disease | 29 days | Grade 2 | Not recovered/not resolved |  |
| 12 to <18 years | Contusion | 4 days | Grade 1 | Recovered/resolved  (duration = 11 days) | *–* |
|  | Gastroenteritis viral | 64 days | Grade 1 | Recovered/resolved  (duration = 1 days) | – |
|  | Hepatic fibrosis^d^ | 176 days | Grade 2 | Not recovered/not resolved | *Current:* congenital heart disease  *Past:* cardiac procedure for congenital heart disease |
|  | Worsening of congenital heart disease^d^ | 191 days | Grade 1 | Recovering/resolving |  |

Abbreviation: AE, adverse event.

^a^None of the post-dose AEs’ were considered to be treatment-related according to the investigator

^b^Standard toxicity grading according to the DAIDS Table for Grading the Severity of Adult and Pediatric Adverse Events, version 2.1 (July 2017)

^c^All AEs in this age group were reported in the same participant.

^d^AEs were reported in the same participant.

## Supplementary Table 6. Absolute and Change from Baseline in Mean Viral Load (Log_10_ Copies/mL) in Nasal Secretions Measured by qRT-PCR

| Visit |  | Sotrovimab IV  6 to <12 years (n = 3) | | Sotrovimab IV  12 to <18 years (n = 5) | | Sotrovimab IV  Total (n = 8) | |
| --- | --- | --- | --- | --- | --- | --- | --- |
|  |  | Absolute viral load (log_10_ copies/mL) | Change from baseline viral load (log_10_ copies/mL) | Absolute viral load (log_10_ copies/mL) | Change from baseline viral load (log_10_ copies/mL) | Absolute viral load (log_10_ copies/mL) | Change from baseline viral load (log_10_ copies/mL) |
| Baseline | n | 2 | – | 5 | – | 7 | – |
|  | Mean (SD) | 5.085 (0.248) | – | 4.712 (1.067) | – | 4.819 (0.896) | – |
| Day 3 | n | 3 | 2 | 5 | 5 | 8 | 7 |
|  | Mean (SD) | 4.477 (2.717) | -2.110 (0.863) | 3.662 (1.137) | -1.050 (1.485) | 3.968 (1.739) | -1.353 (1.365) |
| Day 5 | n | 3 | 2 | 5 | 5 | 8 | 7 |
|  | Mean (SD) | 4.097 (2.093) | -1.270 (2.630) | 2.552 (0.725) | -2.160 (1.405) | 3.131 (1.480) | -1.906 (1.631) |
| Day 8 | n | 3 | 2 | 5 | 5 | 8 | 7 |
|  | Mean (SD) | 3.567 (2.053) | -2.640 (0.693) | 2.314 (0.753) | -2.398 (1.627) | 2.784 (1.396) | -2.467 (1.364) |
| Day 11 | n | 3 | 2 | 5 | 5 | 8 | 7 |
|  | Mean (SD) | 2.940 (2.009) | -3.305 (0.248) | 1.842 (0.139) | -2.870 (1.143) | 2.254 (1.220) | -2.994 (0.962) |
| Day 29 | n | 3 | 2 | 5 | 5 | 8 | 7 |
|  | Mean (SD) | 1.780 (0.000) | -3.305 (0.248) | 1.780 (0.000) | -2.932 (1.067) | 1.780 (0.000) | -3.039 (0.896) |

Abbreviations: IV, intravenous; qRT-PCR, quantitative reverse transcriptase polymerase chain reaction; SD, standard deviation.

Nasal mid-turbinate swabs were taken on Days 1, 3, 5, 8, 11, and 29 for virology analyses. Viral-load data were compared with baseline. Participants with an increase of >1 log_10_ copies/mL in viral load at any point following any previous sample, or whose viral load became quantifiable after having been below the lower limit of detection or quantification, were considered as meeting the criteria for viral rebound. Negative and <2.08 log_10_ copies/mL viral-load samples are imputed as 1.78 log_10_ copies/mL. Baseline is defined as the latest non-missing value prior to dosing. One participant was missing a quantifiable sample at baseline and is not included in the baseline viral load or change from baseline viral load summaries.

### **Supplementary Figure 1.** Study design.


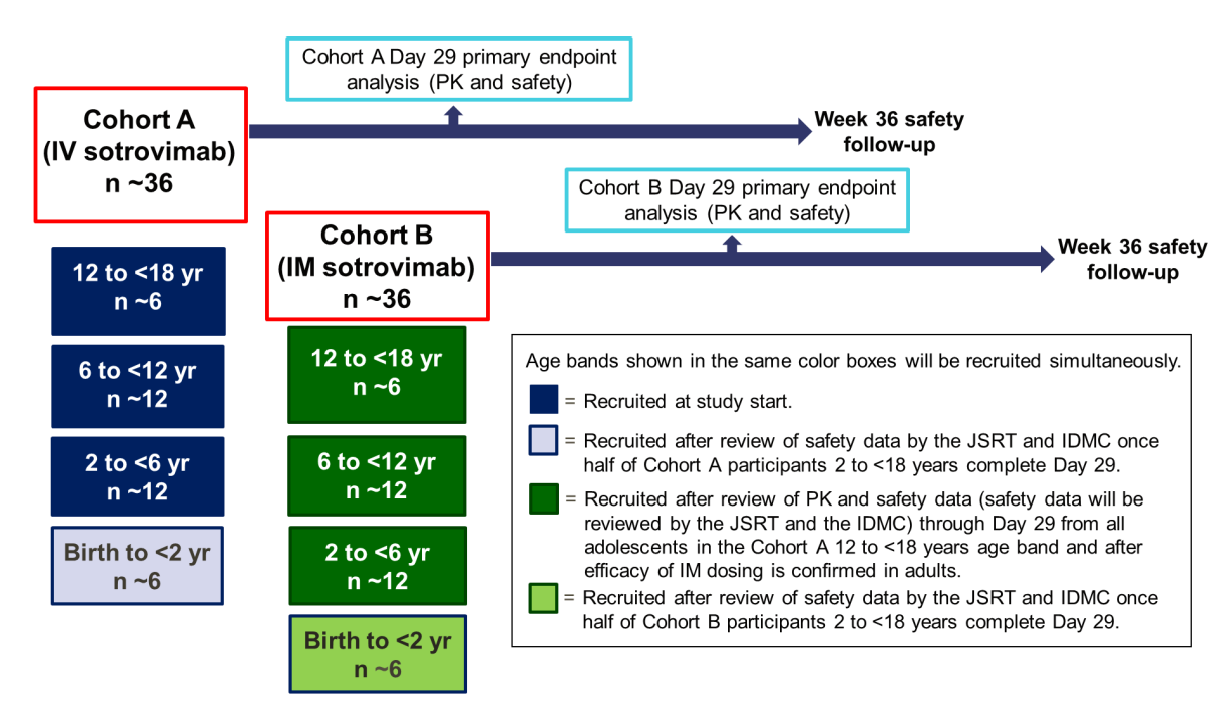


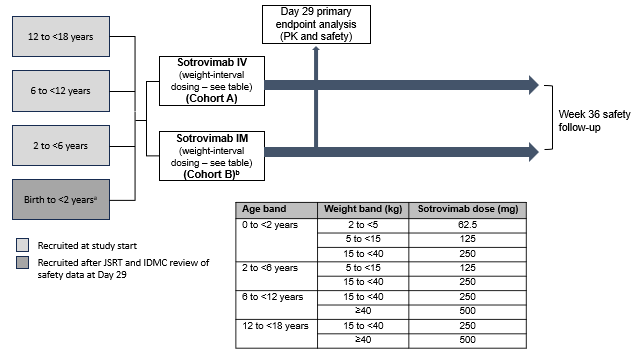


Abbreviations: IDMC, Independent Data Monitoring Committee; IM, intramuscular; IV, intravenous; JSRT, Joint Safety Review Team; PK, pharmacokinetics.

In Cohort A, no participants were enrolled in the 2 to <6 years and birth to <2 years age groups due to early termination of enrollment.

Cohort B (sotrovimab IM administration) was not initiated due to early termination of enrollment.

Sotrovimab was planned to be administered undiluted using a syringe pump for participants weighing 2–<15 kg, and diluted in 40 mL saline for those weighing ≥15 kg.

### **Supplementary Figure 2.** Participant disposition.


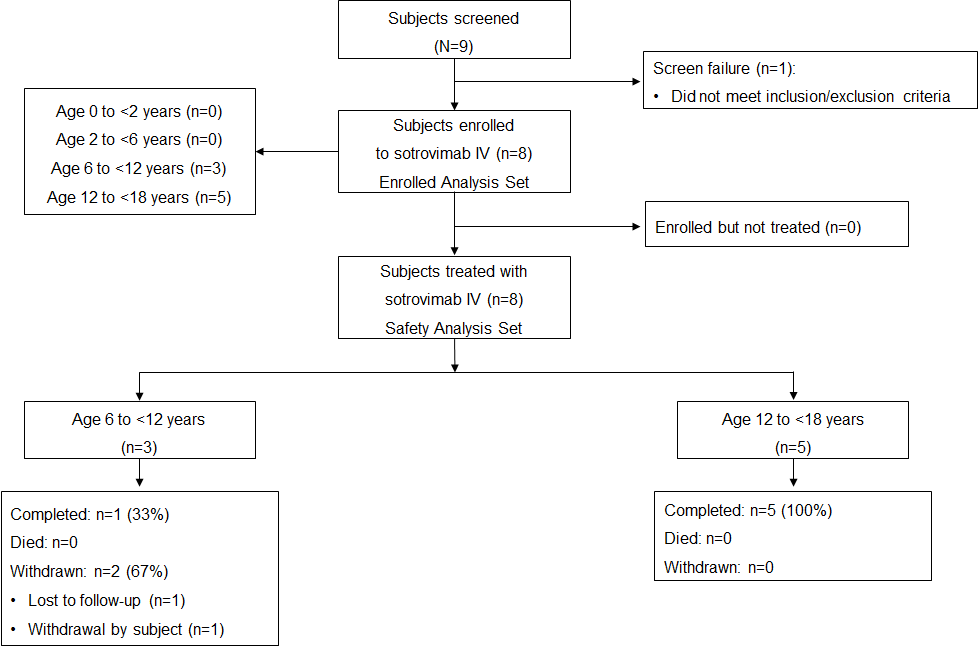


Abbreviation: IV, intravenous.

## Supplementary Figure 3. Viral-load profiles for pediatric participants post-sotrovimab administration (virology analysis set).


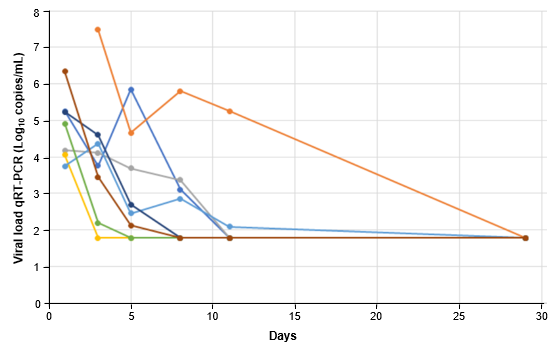


Abbreviation: qRT-PCR, quantitative reverse transcriptase polymerase chain reaction.

Data show individual viral-load profiles from the total population (N = 8).

Negative and <2.08 log_10_ copies/mL viral-load samples were imputed as 1.78 log_10_ copies/mL. Baseline was defined as the latest non-missing value prior to dosing. One participant was missing a quantifiable sample at baseline.
